# Supplementary material for: Hybrid Models and Biological Model Reduction with PyDSTool
Source: PLoS Comput Biol. 2012 Aug 9;8(8):e1002628. doi: 10.1371/journal.pcbi.1002628 (PMC3415397; doi:10.1371/journal.pcbi.1002628)
Supplement: Text S4 — Complete source code for the PyDSTool package (version 0.88.120504). Includes API documentation and help files linking to web pages. This file is identical to the current public release on Sourceforge.net. (ZIP) [file pcbi.1002628.s004.zip › PyDSTool/html/PyDSTool.Toolbox.optimizers.line_search.goldstein_rule-pysrc.html]

xml version="1.0" encoding="ascii"?


PyDSTool.Toolbox.optimizers.line\_search.goldstein\_rule


| Home | Trees | Indices | Help | PyDSTool |
| --- | --- | --- | --- | --- |

Package PyDSTool ::
Package Toolbox ::
Package optimizers ::
Package line\_search ::
Module goldstein\_rule

[hide private]

[frames] | no frames]

# Source Code for Module PyDSTool.Toolbox.optimizers.line\_search.goldstein\_rule

```
 1   
 2  # Matthieu Brucher 
 3  # Last Change : 2007-08-22 14:04 
 4   
 5  import numpy 
 6   


7 -class GoldsteinRule(object):


8    """ 
 9    The Goldstein rule for a inexact line search 
10    """ 


11 -  def __init__(self, rho = 0.1, alpha_min = 0., alpha_max = 1., **kwargs):


12      """ 
13      Initializes the search 
14      Parameters : 
15        - rho is the factor 
16        - alpha_min is the inf limit of the search interval (0.) 
17        - alpha_max is the max limit of the search interval (1.) 
18      Those parameters should be tweaked depending on the function to optimize 
19      """ 
20      self.rho = rho 
21      self.alpha_min = alpha_min 
22      self.alpha_max = alpha_max

23   


24 -  def __call__(self, origin, function, state, **kwargs):


25      """ 
26      Tries to find an acceptable candidate 
27      """ 
28      direction = state['direction'] 
29      gradient = state['gradient'] 
30      ak = self.alpha_min 
31      if 'initial_alpha_step' in state: 
32        bk = state['initial_alpha_step'] 
33      else: 
34        bk = self.alpha_max 
35      alpha = (ak + bk)/2. 
36   
37      f1temp = function(origin) 
38      ft = function(origin) 
39      while(True): 
40        #print ak, bk, alpha 
41        if numpy.isnan(alpha): 
42          state['alpha_step'] = 0 
43          return origin 
44   
45        ftemp = function(origin + alpha * direction) 
46        #First rule test 
47        if ftemp <= ft + self.rho * alpha * numpy.dot(gradient, direction): 
48   
49          #Second rule test 
50          if ftemp >= ft + (1 - self.rho) * alpha * numpy.dot(gradient, direction): 
51            state['alpha_step'] = alpha 
52            return origin + alpha * direction 
53          else: 
54            ak = alpha 
55            alpha = (ak + bk)/2. 
56        else: 
57          bk = alpha 
58          alpha = (ak + bk)/2.

59
```

  


| Home | Trees | Indices | Help | PyDSTool |
| --- | --- | --- | --- | --- |

|  |  |
| --- | --- |
| Generated by Epydoc 3.0.1 on Fri May 4 15:24:24 2012 | http://epydoc.sourceforge.net |
